# Quantum computing for several AGV scheduling models

**DOI:** 10.1038/s41598-024-62821-6

**Published:** 2024-05-28

**Authors:** Liang Tang, Chao Yang, Kai Wen, Wei Wu, Yiyun Guo

**Affiliations:** 1 College of Transportation Engineering, Dalian Maritime University, Dalian, China; 2 Beijing QBoson Quantum Technology Co., Ltd, Beijing, China; 3 Faculty of Engineering, Shizuoka University, Hamamatsu, Japan; 4 Department of Information Science and Engineering, Ocean University of China, Qingdao, China

**Keywords:** Automated guided vehicles, Scheduling, Quantum computing, Quadratic unconstrained binary optimization, Coherent Ising machine, Applied mathematics, Computer science, Quantum physics

## Abstract

Due to the high degree of automation, automated guided vehicles (AGVs) have been widely used in many scenarios for transportation, and traditional computing power is stretched in large-scale AGV scheduling. In recent years, quantum computing has shown incomparable performance advantages in solving specific problems, especially Combinatorial optimization problem. In this paper, quantum computing technology is introduced into the study of the AGV scheduling problem. Additionally two types of quadratic unconstrained binary optimisation (QUBO) models suitable for different scheduling objectives are constructed, and the scheduling scheme is coded into the ground state of Hamiltonian operator, and the problem is solved by using optical coherent Ising machine (CIM). The experimental results show that compared with the traditional calculation method, the optical quantum computer can save 92% computation time on average. It has great application potential.

## Introduction

The scale of the logistics industry has maintained a considerable growth rate, and many human and material resources have been invested in it, thus creating the labor-intensive industry. Improving the automatic and intelligent level of the logistics industry has become an important issue for industry and academia. In recent years, some of the industry’s leading enterprises have already carried out technological reforms. For example, the retail giant, Amazon, has established a huge logistics center, that uses intelligent sorting technology, delivery drones, automated guided vehicles (AGVs), etc. China’s e-commerce giant, Jingdong, has also set up an ‘Asia One’ warehouse, in which more than 100 AGVs are used for transportation operations at the same time^1^. In addition, technical innovation also occurred in ports. In 1993, the world’s first automated wharf was built in the Amsterdam Port in the Netherlands, and dozens of AGVs were used for container transshipment. With the introduction of technology and the accumulation of operation experience, the construction of automatic terminals has been tried all over the world, and the usage of AGVs has gradually increased. Currently, the use of AGVs has penetrated all aspects of logistics, transportation and production, which has greatly promoted the level of industrial automation and intelligence and improved efficiency.

To meet the needs of application scenarios, the amount of parallel work of AGVs is increasing, which brings great difficulty to the AGV scheduling. AGV scheduling problem is a difficult combinatorial optimization problem, and a large number of researchers have devoted themselves to this field and made some contributions. Singh et al.^2^ considered AGV scheduling with battery constraints, developed a mixed-integer programming model with the objective of minimizing the combined task delay cost and AGV transportation cost, and designed a customized adaptive large-neighborhood search algorithm to solve the model. Zhang et al.^3^ studied an AGV scheduling problem in matrix manufacturing plants, and proposed a mixed integer programming model to minimize the generalized transportation cost, based on which an improved iterative greedy algorithm was designed and compared with six other algorithms to show its superior solution performance. For the scheduling problem of AGVs in smart factories, Zhang et al.^4^ proposed a self-organized dynamic scheduling method, that groups multiple AGVs to perform tasks among themselves and uses improved gene expression programming to learn dynamic scheduling rules. The numerical experimental results show that the method can considerably reduce system costs. Wang and Zeng^5^ studied the port AGV scheduling and path planning problem under conflict-free paths, established a mixed integer model with the objective of minimizing task completion time, and proposed a customized branch and bound algorithm combined with a heuristic algorithm to solve the small-scale problem, and further developed a two-stage greedy heuristic algorithm to quickly obtain a satisfactory solution for the large-scale problem. Sagar and Jerald^6^ proposed a real-time scheduling strategy for AGVs based on deep reinforcement learning technology, established a Markov decision model for real-time scheduling, and developed a Q-learning algorithm. The superiority of the method is shown through numerical experiments. Considering the scheduling and path planning model of shop floor AGVs, Saidi et al.^7^ developed a discrete-time model and proposed a two-stage ant colony algorithm to solve the model. From the above literature, the research on scheduling problems of AGVs covers several scenarios such as workshops and terminals. Researchers have built mixed integer programming models, integer programming models, Markov decision process models, etc. The methods used are scheduling rules, exact algorithms, heuristic algorithms, reinforcement learning algorithms, etc. From the results, it is observed that the exact algorithm can generate optimal scheduling solutions, however, its computational time is prohibitively slow, rendering it impractical for large-scale problems. Inexact algorithms exhibit favorable efficiency but often converge to local optima. The provision of high-quality scheduling solutions within a short timeframe poses a significant challenge.

In recent years, significant advancements have been made in both theoretical understanding and practical applications of quantum computing. The fundamental distinction between quantum computers and traditional computers lies in their reliance on quantum mechanical principles. Quantum computers utilize quantum bits (qubits) as the fundamental units of information storage^8^, which can exist in superposition states of both 1 and 0, enabling them to hold exponentially more information compared to traditional computers. It is well recognized that quantum computers offer substantial advantages, particularly in addressing specific problems such as combinatorial optimization, often described as the superiority of quantum computing. Many combinatorial optimization problems are NP-hard, presenting significant challenges for traditional computers to solve. Combinatorial optimization problem can be mapped to the ground state search problem of Ising model. Hardware systems can be built in many different ways to simulate the process of Hamiltonian reduction, such as adiabatic quantum computing (AQC), quantum annealing (QA), etc. However, it is always a difficult problem to improve the connection density between qubits, which will affect the efficiency of problem solving^9,10^. Coherent Ising Machine (CIM) is a quantum computer developed according to the optical principle^11–17^, which can work at room temperature and deal with large-scale problems, such as compression sensor problems^18^ and polyhedron problems^19^. CIM uses laser pulses in optical fiber as qubits for quantum calculation. The early prototype of CIM is injection synchronous laser Ising machine. The number of coupled lasers in this scheme is proportional to the square of qubits, which is quite difficult. On this basis, optical delay linear CIM and measurement feedback CIM using nonlinear optical crystal instead of laser are developed. The latter uses measurement feedback to avoid the challenge that the former needs to control a large number of optical delay lines accurately^14^. The machine used in this study is measurement feedback CIM.

AGV scheduling problem can be understood as a kind of routing problem. Most traditional solutions to routing problems require sacrificing large amounts of computational resources and Osaba et al.^20^ indicated that quantum computing techniques have great potential in the area of solving routing and optimization problems. In the early days, Goswami et al.^21^ developed a phase estimation technique to solve the traveling salesman problem (TSP), using IBM’s quantum simulator to provide results for four city cases. Then, researchers tried to solve more complex problems with quantum computing. Feld et al.^22^ presented a quadratic unconstrained binary optimization (QUBO) formulation for solving the vehicle routing problem with capacity constraints, evaluated the solution quality and computation time and compared it with classical solution methods. Bao et al.^23^ proposed a two-stage QUBO formulation of the vehicle routing problem with balanced pickup, mapping the first stage to a clustering problem and describing the second stage as a TSP problem, and evaluated it against traditional methods in terms of numerical experimental results. Harwood et al.^24^ tried to establish a qubo model to describe the vehicle routing problem by using the modeling idea of node and arc, and evaluated the model by using analog quantum devices. Geitz et al.^25^ built a QUBO model to solve the job-shop scheduling problem, using quantum computers or simulators, constrained programming and tabu search. The calculation results proved the effectiveness of quantum computing in small-scale situations. And the established QUBO model can be extended to AGV scheduling problem. Ohzeki et al.^26^ formulated an Ising model for the collision-free scheduling problem of AGVs within a factory setting. They utilized a quantum annealing machine to solve the model, with results demonstrating the potential application of quantum annealing machines in addressing real-world industrial challenges. Based on the above cases, it can be seen that some researchers have begun to use quantum computing to solve practical problems in the field of optimization. However, the research on quantum computing related to AGV scheduling has just started, and many researchers used simulators to solve them, because the current physical real machine resources are scarce, and the scale of solving problems is still relatively small, and it is easy to make mistakes and lacks the running data of physical real machines.

### Our contributions

In a word, most of the existing AGV scheduling research adopts traditional models and methods, which can not effectively meet the actual needs of large-scale scheduling. Quantum computing has great application potential in solving specific problems that traditional computers cannot solve, and researchers have tried in optimization fields. However, as far as we know, there are few literatures about AGV scheduling using quantum computing technology. Based on these facts, the idea of carrying out this research came into being. The main contributions of this paper are summarized as follows.

1. In traditional research on the AGV scheduling problem, the computation time increases greatly with an increase in the number of AGVs and tasks. We introduce quantum computing technology into the research of the AGV scheduling problem and construct new QUBO models of AGV scheduling. In real scenarios, dispatchers often set different scheduling objectives according to the nature of the work, among which minimizing the total AGV travel time and minimizing the task completion time (makespan) are the two most common objectives. According to the different objectives, we have deduced different QUBO models, and given the model solutions and related theoretical basis under two different objectives.
2. We use traditional computer and CIM to carry out numerical experiments on the traditional model and QUBO model proposed by us respectively. The experimental results show that the computation speed of CIM is much faster than that of traditional computer, and the average calculation time is saved by 92%, which proves that CIM has great application potential in solving AGV scheduling problem and similar combinatorial optimization problems.

## AGV scheduling model

AGV scheduling problems have many classifications according to different scenarios and considerations. For example, consider the time window of the task, joint optimization of scheduling and path, cooperation with other devices, charging strategy and so on. Due to the limitation of quantum bits of CIM, it is impossible to solve the AGV scheduling problem in complex scenes^27–29^. Therefore, we simplify the problem and keep the essence of AGV scheduling problem. On this basis, we construct the AGV scheduling model. In this section, we present the classical AGV scheduling model based on mixed integer programming (MIP), and propose two new models, which we call the node and arc models.

### Problem description

**Figure 1:**
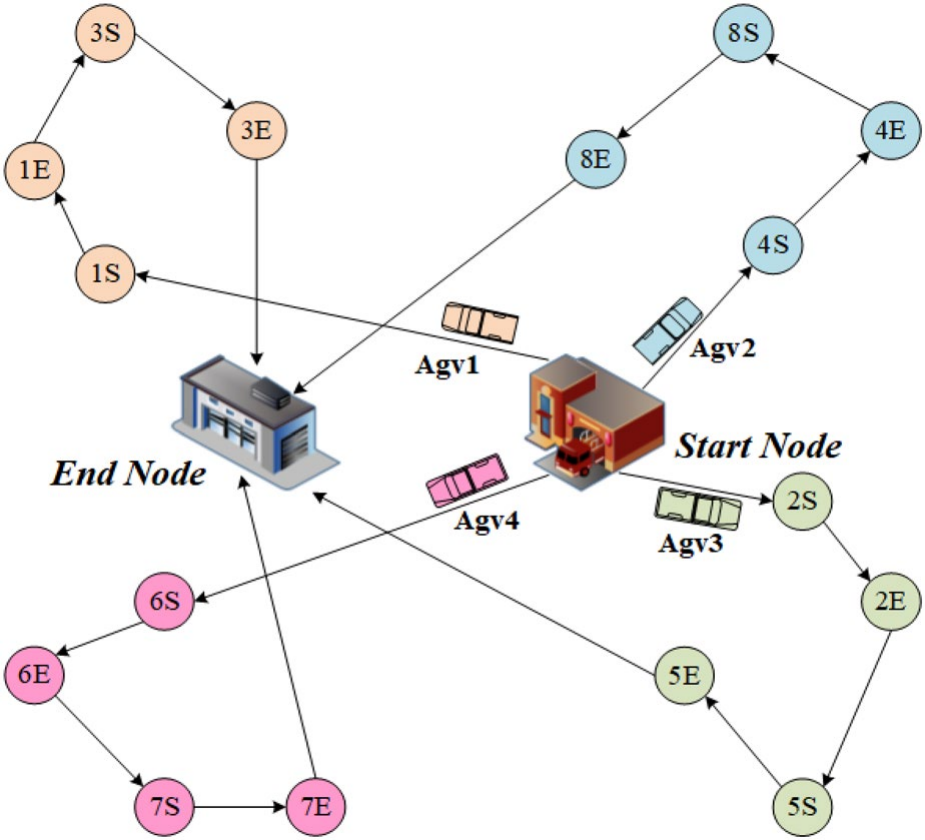
The AGV scheduling problem and a feasible solution. All AGVs start from a fixed start node, perform transportation tasks, and reach the end node after performing all tasks. ’S’ represents the starting point of a transportation task, and ’E’ represents the end point of a transportation task. Different colors represent different AGV’s mission routes.

We consider an AGV scheduling problem (to make the problem more general, we do not set up a working scenario), as shown in Fig. 1. Given an AGV set, all AGVs have a unified starting node and ending node, and all AGVs start to accept tasks from the unified starting node until all transportation tasks are completed, and then return to the unified ending node. In the AGV scheduling problem, we are given a set of transportation tasks, each with a starting point and an ending point. For an AGV, the process of completing the transportation task can be described as first arriving at the starting point of the task to load the transported goods, then transporting them to the ending point of the task, and then driving to the starting point of the next task to perform the next task. The travel time of the AGV between any two task points is known. We consider two optimization objectives, the first is to minimize the total AGV travel time and the second is to minimize the maximum task completion time (makespan). The first objective is generally used when the task is not urgent to achieve a reduction in total system energy consumption, while the second objective is set to complete transportation tasks quickly.

Next, we elaborate on the symbolic settings in the problem as follows.

$V=\{1,\ldots ,k,\ldots ,K \}$ set of AGVs,

$R=\{1,\ldots ,r,{{r}^{'}},{{r}^{''}},\ldots ,n-1 \}$ set of actual tasks,

${{R}_{1}}=\{0,1,\ldots ,r,{{r}^{'}},{{r}^{''}},\ldots ,n-1\}$ set of actual tasks and virtual start task,

${{R}_{2}}=\{1,\ldots ,r,{{r}^{'}},{{r}^{''}},\ldots ,n\}$ set of actual tasks and virtual end task,

${{R}^{'}}=\{0,1,\ldots ,r,{{r}^{'}},{{r}^{''}},\ldots ,n\}$  set of all tasks,

$A=\{(r,{{r}^{'}})\mid r,{{r}^{'}}\in {{R}^{'}}\}$ arc set that consists of all valid task pairs that can be conducted adjacently,

$\pi =\{1,\ldots ,t,\ldots ,N \}$ set of the sequence of tasks performed by an AGV

*a*        a single task arc

$\theta _{r}^{+}$      an arc with task *r* as the left node

$\theta _{r}^{-}$      an arc with task *r* as the right node

$r_{\text{s}}$       starting point of task *r*,

$r_{\text{d}}$       ending point of task *r*.

${{e}_{{{r}_\text{s}}{{r}_\text{d}}}}$     indicates the travel time of the AGV from two points ${{r}_\text{s}}$ and ${{r}_\text{d}}$,

${{e}_{{{r}_\text{d}}r_\text{s}^{'}}}$     indicates the travel time of the AGV from two points ${{r}_\text{d}}$ and $r_\text{s}^{'}$,

${{c}_{r{{r}^{'}}}}$      contains two parts of time, the first part is the time from the start of task *r* to the end of task *r*, and the second part is the time from the end of task *r* to the start of task ${r}^{'}$, ${{c}_{r{{r}^{'}}}}={{e}_{{{r}_{s}}{{r}_{d}}}}+{{e}_{{{r}_{d}}r_{s}^{'}}}$.

### Mixed integer programming model

In this subsection, we introduce the classical model of AGV scheduling. The classical model is formed as a mixed integer programming, and it has the following variables.

${{y}_{r,{{r}^{'}},k}}$       binary variable, equal to 1 if task *r* is performed directly prior to ${{r}^{'}}$ by AGV *k*, 0 otherwise;

$f^\text{s}_{rk}$          arrival time of AGV *k* at the start node of task *r*;

$f^\text{d}_{rk}$          arrival time of AGV *k* at the end node of task *r*;

*T*           the makespan for AGVs to perform transportation tasks.

The first optimization objective of the MIP model is to minimize the total AGV travel time, and its model is presented as follows.

1 $$\begin{aligned} \text {(P1)}\qquad&\text {min}{} & {} \sum _{k\in V} f_{nk}^\text{d} \end{aligned}$$

2 $$\begin{aligned}&\text {s.t.}{} & {} \sum _{r'\in R_2} y_{0,r',k}=1&\forall k\in V \end{aligned}$$

3 $$\begin{aligned}{} & {} {}&\sum _{r\in R_1}y_{r,n,k}=1&\forall k\in V \end{aligned}$$

4 $$\begin{aligned}{} & {} {}&\sum _{r'\in R_2}\sum _{k\in V} y_{r,r',k}=1&\forall r\in R \end{aligned}$$

5 $$\begin{aligned}{} & {} {}&\sum _{r'\in R_1}y_{r',r,k}-\sum _{r'\in R_2} y_{r,r',k}=0&\forall r\in R,\forall k\in V \end{aligned}$$

6 $$\begin{aligned}{} & {} {}&f_{0k}^\text{s}=f_{0k}^\text{d}=0&\forall k\in V \end{aligned}$$

7 $$\begin{aligned}{} & {} {}&f_{rk}^\text{s}+e_{r_\text{s}r_\text{d}}=f_{rk}^\text{d}&\forall k\in V, \forall r\in R' \end{aligned}$$

8 $$\begin{aligned}{} & {} {}&f_{rk}^\text{d}+e_{r_\text{d}r'_\text{s}}\le f_{r'k}^\text{s}+M(1-y_{r,r',k})&\forall r,r'\in R,\forall k\in V \end{aligned}$$

9 $$\begin{aligned}{} & {} {}&y_{r,r,k}=0&\forall r\in R,\forall k\in V \end{aligned}$$

10 $$\begin{aligned}{} & {} {}&{{y}_{r,{{r}^{'}},k}}\in \{0,1\}\text { }&\forall {r,}{{{r}}^{'}}\in {{R}^{'}},\forall k\in V \end{aligned}$$

11 $$\begin{aligned}{} & {} {}&f_{rk}^{s},f_{rk}^{d}\ge 0\text { }&\forall {r}\in {R,}\forall {k}\in {V}. \end{aligned}$$

The objective function (1) is to minimize the total travel time of the AGV. Constraints (2) and (3) ensure that all AGVs need to complete the virtual start task and the virtual end task, and constraints (4) guarantee that all actual tasks are uniquely assigned to a particular AGV. Constraints (5) ensure that each AGV completes its task satisfying the flow balance. Then, constraints (6) guarantee that the virtual start task starts and ends at moment 0. Constraints (7) states that the time to reach the end of a task is equal to the time to reach the start of that task plus the transport time from the start to the end. Constraints (8) indicates that the time to reach the start of a task is later than the time to reach the end of the previous task plus the transportation time required to reach the start of that task from the end of the previous task, where *M* is a sufficiently large value. The last constraints (9) eliminates task self-citation. Constraints (10) and (11) represent range limits of variables.

A slight modification of the above model can be used as a model for minimizing the makespan for AGVs to perform transportation tasks, which is given as follows, Eqs. (2)–(11).

12 $$\begin{aligned} \text {(P2)}\qquad&\text {min}{} & {} T \end{aligned}$$

13 $$\begin{aligned}&\text {s.t.}{} & {} T\ge f_{nk}^{d}&\forall k\in V. \end{aligned}$$

where *T* denotes the makespan. The objective function is to minimize *T*, and constraints (13) denotes that *T* must be no less than the time required by the last AGV to complete the task.

### QUBO model

#### QUBO and Ising model

This section mainly describes the concepts of QUBO model and Ising model and their relationship. QUBO is an expression of optimization problem, and its goal is to find binary variables that minimize quadratic polynomials. Ising model was first put forward and applied in statistical physics. It describes a system composed of interacting units, in which each spin particle must have two possible random states (such as + 1 and − 1), and then it was introduced into the field of mathematics as a model to describe a series of optimization problems. Many combinatorial optimization problems can be expressed in the form of quadratic unconstrained binary optimization or ising model, and they can be transformed into each other^30–32^. The general expression of QUBO model is shown in Eq. (14).

14 $$\begin{aligned} H(x)={{x}^\texttt{T}}Qx+{{c}^\texttt{T}}x,x\in {{\{0,1\}}^{z}} \end{aligned}$$

where *x* is a *z* dimensional vector of binary variables, *Q* is the quadratic coefficient matrix, and ${{c}^\texttt{T}}$ is the coefficient matrix of the primary term.

The above model in the form of QUBO can be easily transformed into an Ising model, and the variable range of the Ising model is $\left\{ -1,1 \right\}$. Specifically, it can be realized by variable substitution ${{\sigma }_{i}}=2{{x}_{i}}-1$. Then, the optimization function can be expressed in the following form.

15 $$\begin{aligned} H(\sigma )=-\sum \limits _{i,j}{{{J}_{ij}}}{{\sigma }_{i}}{{\sigma }_{j}}-\sum \limits _{i}{{{h}_{i}}}{{\sigma }_{i}} \end{aligned}$$

where the ${{\sigma }_{i}}$ is spin variable, ${{J}_{ij}}$ and ${{h}_{i}}$ are the quadratic and linear coefficients. The solution of Ising problem is to find the ground state of Hamiltonian. CIM solves the Ising problem according to the principle of minimum gain, and can find the ground state or low energy state of Ising Hamiltonian. The method is to map the QUBO problem into a fully connected Ising Hamiltonian with programmable parameters, and obtain the solution of the problem through controllable quantum phase transition^33,34^.

#### Node model

In this section, we will describe the node model. The core idea of node model is to regard tasks as nodes and the order of task execution as the order of vehicles passing through nodes.The node model has the QUBO form and is suitable for quantum computing, the variables of the model are described as below.

${{x}_{r,t,k}}$ binary variable, equal to 1 if task *r* is assigned to AGV *k* as it is *t*-th task, 0 otherwise.

The first optimization objective of the node model is to minimize the total AGV travel time, and the model is shown below.

16 $$\begin{aligned} \text {(P3)}\qquad {{H}_{1}}&={{\partial }_{1}}{{H}_{A}}+{{\partial }_{2}}{{H}_{B}}+{{\partial }_{3}}{{H}_{C}}+{{\partial }_{4}}{{H}_{D}}+{{\partial }_{5}}{{H}_{E}}+{{\partial }_{6}}{{H}_{F}} \end{aligned}$$

17 $$\begin{aligned} {{H}_{A}}&=\sum \limits _{k\in V}{\sum \limits _{(r,{{r}^{'}})\in A}{\sum \limits _{t=1}^{N-1}{{{c}_{r{{r}^{'}}}}}}}{{x}_{r,t,k}}{{x}_{{{r}^{'}},t+1,k}} \end{aligned}$$

18 $$\begin{aligned} {{H}_{B}}&={{\sum \limits _{k\in V}{(1-{{x}_{0,1,k}})}}^{2}}\ \end{aligned}$$

19 $$\begin{aligned} {{H}_{C}}&=\sum \limits _{k\in V}{\sum \limits _{r\in R}{\sum \limits _{t=2}^{N-1}{{{x}_{n,t,k}}}}}{{x}_{r,t+1,k}} \end{aligned}$$

20 $$\begin{aligned} {{H}_{D}}&=\sum \limits _{r\in R}{{{\left( 1-\sum \limits _{k\in V}{\sum \limits _{t=2}^{N-1}{{{x}_{r,t,k}}}}\right) }^{2}}} \end{aligned}$$

21 $$\begin{aligned} {{H}_{E}}&=\sum \limits _{k\in V}{\sum \limits _{\begin{array}{c} r,{{r}^{'}}\in {{R}^{'}} \\ r\ne {{r}^{'}} \end{array}}{\sum \limits _{t=1}^{N}{{{x}_{r,t,k}}{{x}_{{{r}^{'}},t,k}}}}} \end{aligned}$$

22 $$\begin{aligned} {{H}_{F}}&=\sum \limits _{k\in V}{\sum \limits _{t=1}^{N-1}{{{\left( \sum \limits _{r\in {{R}_{1}}}{{{x}_{r,t,k}}}-\sum \limits _{{{r}^{'}}\in {{R}_{2}}}{{{x}_{{{r}^{'}},t+1,k}}}\right) }^{2}}}}.\ \end{aligned}$$

${{\partial }_{i}}$ $(i=1,\ldots ,6)$ are weights correspond to each objective function. The objective function (17) is to minimize the total travel time of all the AGVs. The minimization function (18) and (19) ensures that for each AGV, the virtual start task and the virtual end task must be the first task and the last task, respectively. For each actual task, we want it to be assigned exactly to one AGV, so we add the minimization function (20). We also consider the minimization function (21) in order to make each AGV perform at most one task on each task sequence. For a particular AGV, the order of its tasks must be continuous, based on which we set the minimization function (22).

The model described below is modified from the above model to accommodate the goal of minimizing the makespan.

The least time-consuming task model requires finding the AGV with the longest task execution time and minimizing its execution task time, which leads to inequality constraints, as follows.

23 $$\begin{aligned}&T\ge \sum \limits _{(r,{{r}^{'}})\in A}{\sum \limits _{t=1}^{N-1}{{{c}_{r{{r}^{'}}}}}}{{x}_{r,t,k}}{{{x}_{{{r}^{'}},t+1,k}}}\quad \forall k\in V \end{aligned}$$

24 $$\begin{aligned}&{{H}_{Y}}=T \end{aligned}$$

The objective function (24) is to minimize the makespan *T*.

Then we add slack variables to transform the above inequality constraint into an equation, as follows.

25 $$\begin{aligned}&{{H}_{Z}}={{\sum \limits _{k\in V}{\left( T-\sum \limits _{(r,{{r}^{'}})\in A}{\sum \limits _{t=1}^{N-1}{{{c}_{r{{r}^{'}}}}{{x}_{r,t,k}}{{x}_{{{r}^{'}},t+1,k}}-{{T}_{k}}}}\right) }}^{2}} \end{aligned}$$

26 $$\begin{aligned}&{T=}{{\delta }_{1}}\text {+2}{{\delta }_{2}}\text {+}\ldots \text { +}{{\text {2}}^{m}}{{\delta }_{m}}\ \end{aligned}$$

27 $$\begin{aligned}&{{{T}}_{k}}{=}{{\delta }_{1k}}\text {+2}{{\delta }_{2k}}\text {+}\ldots \text { +}{{\text {2}}^{{{m}^{'}}}}{{\delta }_{{{m}^{'}}k}} \end{aligned}$$

${{T}_{k}}$ $(k\in V)$ is the slack variable, and both *T* and ${{T}_{k}}$ $(k\in V)$ need to be represented by binary variables. ${{\delta }_{i}}$ $(i=1,2,\ldots ,m)$ and ${{\delta }_{ik}}$ $(i=1,2,\ldots ,{m}^{'}, {k}\in {V})$ are the discretized auxiliary variables we introduce, whose number is related to the size of the arithmetic case and needs to be estimated. A large number of auxiliary variables will make the difficulty of solving soar. In general, to represent an integer between 0 and $\varsigma$, $\left[ {{\log }_{2}} \varsigma \right] +1$ discretized auxiliary variables need to be introduced, where $[\varsigma ]$ denotes the largest integer that does not exceed $\varsigma$. Of course, if there are non-integer values introduced in the calculation example, then it is necessary to introduce an approximate representation. First, we introduce the precision matrix as follows:

28 $$\begin{aligned} p=\left[ {{\left( \frac{1}{2} \right) }^{0}},{{\left( \frac{1}{2} \right) }^{1}},{{\left( \frac{1}{2} \right) }^{2}},\ldots \right] . \end{aligned}$$

Then the real numbers ${{\varpi }_{i}}$ $(i=1,\ldots ,L)$ in some interval can be approximated as follows.

29 $$\begin{aligned} {{\varpi }_{i}}\approx {{p}^\texttt{T}}{{b}_{i}}\text {=}\left[ {{\left( \frac{1}{2} \right) }^{0}},{{\left( \frac{1}{2} \right) }^{1}},{{\left( \frac{1}{2} \right) }^{2}},\ldots \right] ^\texttt{T}{{b}_{i}} \end{aligned}$$

where ${{b}_{i}}\in {{\{0,1\}}^{L}}$ $(i=1,\ldots ,L)$. If the error is expressed in terms of $\phi$, the error satisfies the following relation:

30 $$\begin{aligned} \phi \le {{\left( \frac{1}{2}\right) }^{L}}. \end{aligned}$$

In this way, we can rewrite (24) and (25) as follows.

31 $$T={{\delta}_{1}}+2{{\delta}_{2}}+\ldots+{{2}^{m}}{{\delta }_{m}}+{{\left(\frac{1}{2}\right)}^{0}}{{\sigma }_{1}}+{{\left(\frac{1}{2}\right)}^{1}}{{\sigma}_{2}}+\ldots+{{\left(\frac{1}{2}\right)}^{L-1}}{{\sigma }_{L}}$$

32 $${{T}_{k}}={{\delta}_{1k}}+2{{\delta}_{2k}}+\ldots+{{2}^{{{m}^{'}}}}{{\delta }_{{{m}^{'}}k}}+{{\left(\frac{1}{2}\right)}^{0}}{{\sigma }_{1k}}+{{\left(\frac{1}{2}\right)}^{1}}{{\sigma}_{2k}}+\ldots+{{\left(\frac{1}{2}\right)}^{{{L}^{'}}-1}}{{\sigma }_{{{L}^{'}}k}}$$

The positive real numbers can be approximated using integer auxiliary variables. ${{\sigma }_{j}}$ $(j=1,2\ldots ,L)$ and ${{\sigma }_{jk}}$ $(j=1,2,\ldots ,{{L}^{'}},k\in V)$ are used to approximate the decimal part. The number of binary variables used is related to the required approximate accuracy, as shown in Formula (28).

Thus, the model under this objective can be represented as follows, Eqs. (18)–(22), (24)–(25), (31)–(32).

33 $$\text{(P4)}\qquad{{H}_{2}}={{\varepsilon}_{1}}{{H}_{Y}}+{{\varepsilon}_{2}}{{H}_{B}}+{{\varepsilon}_{3}}{{H}_{C}}+{{\varepsilon}_{4}}{{H}_{D}}+{{\varepsilon}_{5}}{{H}_{E}}+{{\varepsilon}_{6}}{{H}_{F}}+{{\varepsilon}_{7}}{{H}_{Z}}$$

In (33), ${{\varepsilon }_{i}}$ $(i=1\ldots ,7)$ are weights for each objectives. The model does not satisfy the QUBO form, because ${{H}_{Z}}$ is a quadrinomial binary polynomial, which needs to be degenerated. Next, we provide some analysis of the ${{H}_{Z}}$. First, to make it easier to show our results, we perform a variable substitution, as follows:

34 $$\begin{aligned} \eta \text { =}\sum \limits _{(r,{{r}^{'}})\in A}{\sum \limits _{t=1}^{N-1}{{{c}_{r{{r}^{'}}}}}}{{x}_{r,t,k}}{{x}_{r,t+1,k}}. \end{aligned}$$

The total number of tasks is *N*, and $\eta$ contains $({{N}^{2}}-3N+3)(N-1)$ monomials. Then ${{H}_{Z}}$ can be expanded as follows.

35 $$\begin{aligned} {{H}_{Z}}=\sum \limits _{k\in V}{({{\eta }^{2}}-2\eta T+2\eta {{T}_{k}}+{{T}^{2}}+T_{k}^{2}-2T{{T}_{k}})} \end{aligned}$$

In the ${{H}_{Z}}$, ${{\eta }^{2}}$ is a quadrinomial binary polynomial, $-2\eta T$ and $2\eta {{T}_{k}}$ are cubic binary polynomials, and ${{T}^{2}}$, $T_{k}^{2}$ and $-2T{{T}_{k}}$ are all quadratic binary polynomials, so we need to reduce ${{\eta }^{2}}$, $-2\eta T$ and $2\eta {{T}_{k}}$. The number of quardrinomial binary monomials in ${{\eta }^{2}}$ is represented by ${{\tau }_{4}}$, and ${{\tau }_{4}}$ is as follows:

36 $$\begin{aligned} {{\tau }_{4}}={{(({{N}^{2}}-3N+3)(N-1))}^{2}}|V|. \end{aligned}$$

Note that

37 $$\begin{aligned} {{x}^{n}}=x (n\in {{\mathbb {Z}}^{+}}),x\in (0,1) \end{aligned}$$

where ${{\mathbb {Z}}^{+}}$ represents the set of all positive integers. Equation (37) indicates that any power of the binary variable itself is equal to itself, and thus, $\tau _{4}^{'}$ quardrinomial monomial can be directly reduced, and its specific number is as follows.

38 $$\begin{aligned} \tau _{4}^{'}=(({{N}^{2}}-3N+3)(N-1))|V| \end{aligned}$$

Therefore, the number of quadrinomial polynomials that truly need to be reduced is ${{\tau }_{4}}-\tau _{4}^{'}$ terms. Looking at the part of cubic polynomial, the number of cubic monomials in ${{H}_{Z}}$ is represented by ${{\tau }_{3}}$, and then ${{\tau }_{3}}$ is as follows:

39 $$\begin{aligned} {{\tau }_{3}}=(m+L+{{m}^{'}}+{{L}^{'}})({{N}^{2}}-3N+3)(N-1)|V|. \end{aligned}$$

Then, the number of all polynomials in ${{H}_{Z}}$ that need to be descended $\tau$ is as follows:

40 $$\begin{aligned} \tau ={{\tau }_{3}}+{{\tau }_{4}}-\tau _{4}^{'}. \end{aligned}$$

At least $\tau$ binary auxiliary variables must be introduced to complete the descending order according to a paper^35^. Due to the number of auxiliary variables introduced, the processing is more complex and it is difficult to calculate using existing quantum computers. So this model will not be introduced so far in this paper.

#### Arc model

In this section we will describe the arc model. The core idea of arc model is that the sequence of tasks before and after execution is regarded as an arc connected between nodes, and building the model with arc as the basic unit can reduce the dimension. The arc model also has a QUBO form with the same parameter settings as the node model, and the decision variables are shown below.

${{\upsilon }_{a,t,k}}$ binary variable, equal to 1 if arc *a* is assigned to AGV *k* in sequence *t*, 0 otherwise.

As with the node model, we first explore the model with the optimization objective of minimizing the total travel time.

41 $$\text{(P5)}\qquad{{H}_{3}}={{\beta }_{1}}{{H}_{G}}+{{\beta }_{2}}{{H}_{J}}+{{\beta }_{3}}{{H}_{L}}+{{\beta }_{4}}{{H}_{M}}+{{\beta }_{5}}{{H}_{O}}+{{\beta }_{6}}{{H}_{Q}}+{{\beta }_{7}}{{H}_{S}}+{{\beta }_{8}}{{H}_{U}}$$

42 $$\begin{aligned} {{H}_{G}}&=\sum \limits _{k\in V}{\sum \limits _{a\in A}{\sum \limits _{t=1}^{N-1}{{{c}_{a}}}}}{{\upsilon }_{a,t,k}} \end{aligned}$$

43 $$\begin{aligned} {{H}_{J}}&={{\sum \limits _{k\in V}{\left( 1-\sum \limits _{a\in \theta _{0}^{+}}{{{\upsilon }_{a,1,k}}}\right) }}^{2}} \end{aligned}$$

44 $$\begin{aligned} {{H}_{L}}&=\sum \limits _{k\in V}{\sum \limits _{{\begin{matrix} a\ne {{a}_{1}} \\ a\in \theta _{n}^{-} \\ {{a}_{1}}\in A \end{matrix}}}{\sum \limits _{t=1}^{N-2}{{{\upsilon }_{a,t,k}}{{\upsilon }_{{{a}_{1}},t+1,k}}}}} \end{aligned}$$

45 $$\begin{aligned} {{H}_{M}}&=\sum \limits _{k\in V}{\sum \limits _{{\begin{matrix} a\ne {{a}_{1}} \\ a\in A \\ {{a}_{1}}\in A \end{matrix}}}{\sum \limits _{t=1}^{N-1}{{{\upsilon }_{a,t,k}}}}}{{\upsilon }_{{{a}_{1}},t,k}} \end{aligned}$$

46 $$\begin{aligned} H_O&= \sum _{r\in R}\left( 1-\sum _{k\in V}\sum _{a\in \theta _r^-}\sum _{t=1}^{N-2}v_{a,t,k}\right) ^2 \end{aligned}$$

47 $$\begin{aligned} H_Q&= \sum _{k\in V}\left( 1-\sum _{a\in \theta _0^+}\sum _{t=1}^{N-2}v_{a,t,k}\right) ^2 \end{aligned}$$

48 $$\begin{aligned} H_S&= \sum _{k\in V}\left( 1-\sum _{a\in \theta _n^-}\sum _{t=2}^{N-1}v_{a,t,k}\right) ^2 \end{aligned}$$

49 $$\begin{aligned} {{H}_{U}}&=\sum \limits _{k\in V}{\sum \limits _{r\in R}{\sum \limits _{t=1}^{N-2}{{{\left( \sum \limits _{a\in \theta _{r}^{-}}{{{\upsilon }_{a,t,k}}}-\sum \limits _{{{a}_{1}}\in \theta _{r}^{+}}{{{\upsilon }_{{{a}_{1}},t+1,k}}}\right) }^{2}}}}}. \end{aligned}$$

${{\beta }_{i}}$ $(i=1,\ldots ,8)$ are weights for the corresponding objective. The objective function (42) is to minimize the total travel time of the AGV. We want all AGVs to be executed in the first order a task arc that starts with a virtual start task, so we add the minimization function (43). The minimization function (44) means that the last task completed by each AGV must be a virtual end task. We want to complete at most one task arc in a certain order of an AGV, so we add the minimization function (45). The minimization function (46) indicates that for each actual task, we want a certain task arc with it as the starting node to be assigned to an AGV in a certain order of completion, and we want the virtual start task to be completed only once for each AGV, so we add the minimization function (47). Similarly, for each AGV, its virtual end task can only be completed once, so we add the minimization function (48). The minimization function (49) ensures that for each AGV, it is must to satisfy the flow balance when performing the task arc.

Next, we show a model with the goal of minimizing the makespan as follows, Eqs. (42)–(49).

50 $$\begin{aligned} \text {(P6)}\qquad {{H}_{4}}&={{\lambda }_{1}}{{H}_{V}}+{{\lambda }_{2}}{{H}_{J}}+{{\lambda }_{3}}{{H}_{L}}+{{\lambda }_{4}}{{H}_{M}}+\nonumber \\&{{\lambda }_{5}}{{H}_{O}}+{{\lambda }_{6}}{{H}_{Q}}+{{\lambda }_{7}}{{H}_{S}}+{{\lambda }_{8}}{{H}_{U}}+{{\lambda }_{9}}{{H}_{W}} \end{aligned}$$

51 $$\begin{aligned} {{H}_{V}}&=T \end{aligned}$$

52 $$\begin{aligned} {{H}_{W}}&=\sum \limits _{k\in V}{{{\left( T-\sum \limits _{a\in A}{\sum \limits _{t=1}^{N-1}{{{c}_{a}}{{\upsilon }_{a,t,k}}}}-{{T}_{k}}\right) }^{2}}}. \end{aligned}$$

## Numerical experiments

In this section, we provide rich numerical experimental results, and research data can be obtained on public databases^36^. In the first subsection we use Gurobi^37^ solver to solve the MIP model proposed above on a traditional computer, and show its computing performance under different problem scales. In the second subsection we use optical quantum computer to solve the problem cases of node model and arc model at different scales. And the computation performance is compared with that of traditional computers.

**Figure 2:**
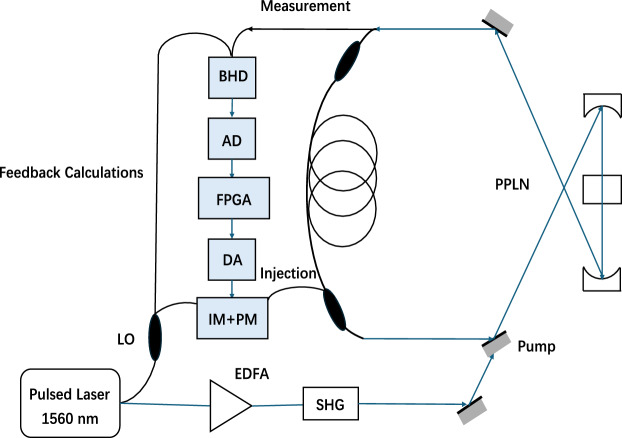
Schematic diagram of coherent Ising mechanism construction and principle.

The CIM we used is provided by Beijing Qboson Quantum Technology Co.Ltd, and its structure and principle diagram are shown in Fig. 2. The components of this CIM are mainly composed of optical parts and electrical parts. The optical part of the machine is composed of pulsed laser, erbium-doped fiber amplifier(EDFA), fiber rings and periodically poled lithium niobate (PPLN) crystals, while the electrical part is mainly composed of optical balanced homodyne detectors (BHD), analog-to-digital/digital-to-analog (AD/DA) converter and field-programmable gate array (FPGA). The laser emits laser with a repetition frequency of 100mhz, which is amplified by EDFA, and then the amplified laser frequency is doubled by PPLN crystal to generate 780 nm laser, which is used as the pump source to synchronously pump the phase sensitive amplifier, forming degenerate optical parametric oscillation(DOPO). There are 211 oscillation pulses in the fiber ring, and the time interval between adjacent pulses is 10 ns, so the transmission time of optical pulses in the ring is 2.11 µs. Then, the laser output in the fiber ring and the laser with the fundamental frequency of 1560 nm are determined by BHD, and the FPGA obtains the feedback signal of the next round trip according to the interaction intensity between spins in Ising Hamiltonian, which is used as the control signal of the intensity modulator (IM), and its sign defines the phase shift (0 or $\pi$) of the phase modulator (PM)^9,14,34,38^.

To compare the performance between CIM and traditional computer, we also run our experiments with Gurobi 9.5.1 on a Mechrevo computer with 2.8 GHz Intel Core i7 CPU and 8GB memory, using up to four threads. The task points used in this experiment are randomly selected on the two-dimensional axis, ranging from 10 to 90, and then Euclidean distance is used as the length between two points, and we design the speed of each AGV to be constant, the time passing through the unit distance is the unit time.

### Computing on a traditional PC

In this section, we use Gurobi to solve the mixed integer programming model of AGV scheduling for two optimization objectives. In “Number of tasks” we show experiments on the variation in computation time with the number of tasks, while in “Number of AGVs” we show experiments on the variation in computation time with the number of AGVs. We set a time limit to 1800 seconds for each run.

#### Number of tasks

**Figure 3:**
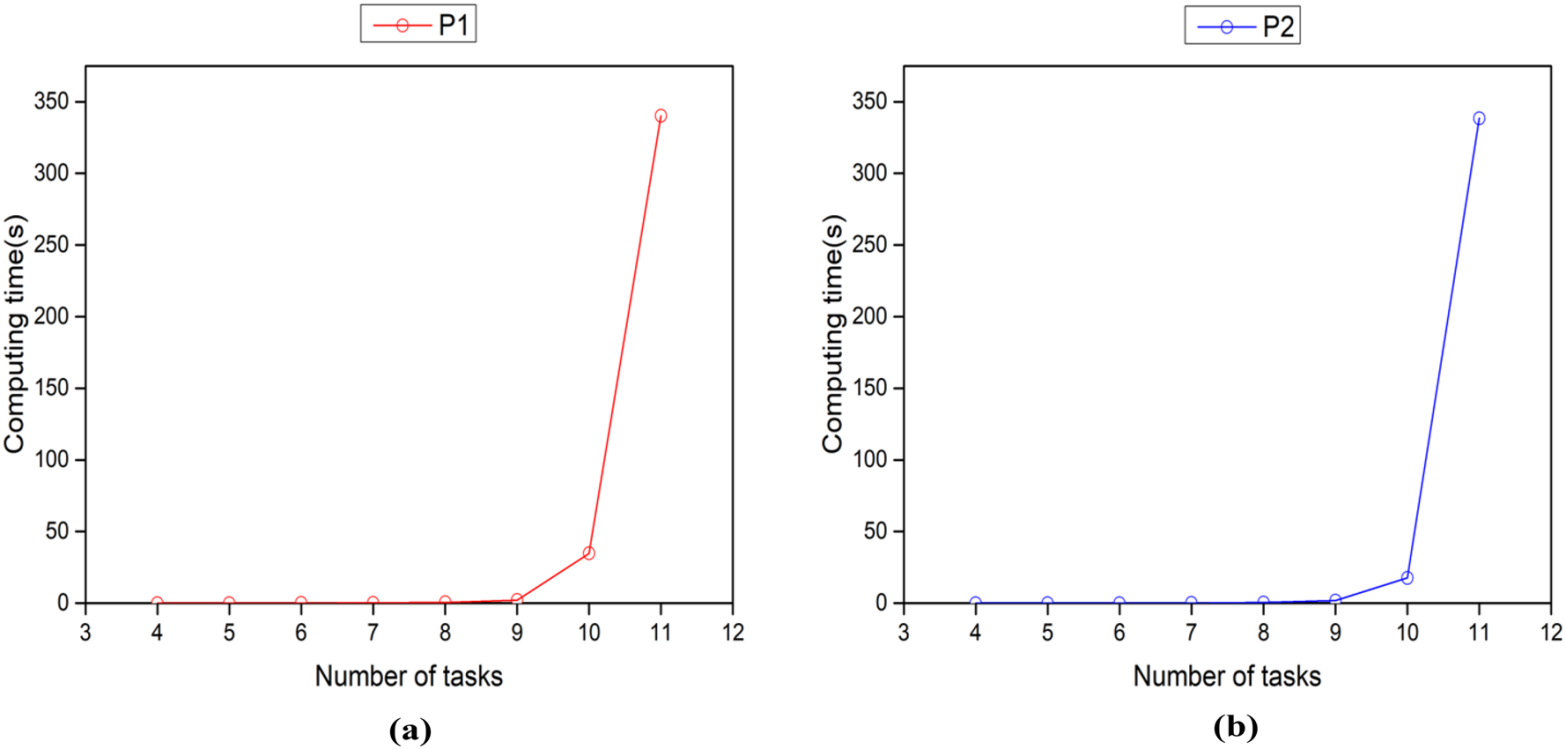
Computation time versus number of tasks for MIP model. (**a**) Represents the change of MIP model computation time with the number of tasks under the goal of minimizing the total travel time. (**b**) Represents the change of MIP model computation time with the number of tasks under the goal of minimizing the makespan.

In general, an increase in the number of tasks leads to a slower generation of AGV scheduling solutions. In this subsection, we investigate the effect of task number variation on the computational speed of the three models proposed in this paper. To achieve this goal, we generate instances of 4 tasks to 12 tasks with a fixed number of AGVs of 2 and obtain the computational time graphs shown in Fig. 3, where the left figure takes the minimum total travel time as the objective function, and the right figure takes the minimum makespan as the objective function. The legend section represents the model number, which corresponds to the previous section number.

In Fig. 3, we find that the computing speed of mixed integer programming model gradually slows down with the increase of the number of AGV tasks, and the computation time increases sharply when the number of tasks reaches a certain critical value, which is a common property reflected by two different objective functions. Especially when the number of tasks increases to 12, the computing time has exceeded 1800s, which reflects the weakness of traditional models in the face of large-scale problems.

#### Number of AGVs

In this subsection, we hope to explore the influence of the change of AGV number on the computing time of mixed integer programming model, so we fixed the number of tasks as 10 and 11, and set the number of AGVs in the range from 2 to 8. We show the results in Tables 1 and 2. Notation “–” in the table implies that the corresponding model failed to obtain an optimal solution within the time limit. An instance with *a* tasks and *b* AGVs are denoted by “a–b”.

**Table 1:** Computation time (s) under objective of total travel time.

instances	MIP
10–2	34.76
10–3	108.30
10–4	150.40
10–5	255.10
10–6	349.00
10–7	398.00
10–8	–
11–2	340.20
11–3	1262.00
11–4	–
11–5	–
11–6	–
11–7	–
11–8	–

**Table 2:** Computation time (s) under objective of makespan.

instances	MIP
10–2	17.75
10–3	28.85
10–4	31.17
10–5	61.01
10–6	58.88
10–7	79.96
10–8	50.79
11–2	338.50
11–3	393.00
11–4	186.70
11–5	204.10
11–6	188.50
11–7	503.20
11–8	405.90

From the results obtained in Tables 1 and 2, we can conclude that there is no strict correlation between the number of AGV and the computing time. Table 1 shows the computational performance of the three models under the objective of minimize the total travel time, and we can observe that the computational performance of the MIP model is very poor, and many groups of experiments failed to obtain an optimal solution within the limited time. Table  2 shows the computational performance of the MIP model under the objective of minimize the makespan, and the model performs much better, the optimal solution is obtained in the limited time in all groups of experiments. However, its computation time is still at a great disadvantage.

### Computational experiment on CIM

In this subsection, we use the CIM to solve the QUBO model and compare its performance with the MIP solver on traditional PC. Since the maximum number of Quantum bits of the CIM used in this research is 100, all the comparative examples in this section limit the number of variables to 100. Based on this, we completed six groups of computation experiments with a quantum computer. In all the experiments, the number of AGV was limited to two. For the numerical experiment of node model, we set the number of tasks to 4 to 7, while for the numerical experiment of arc model, we fixed the number of tasks to 4.

**Figure 4:**
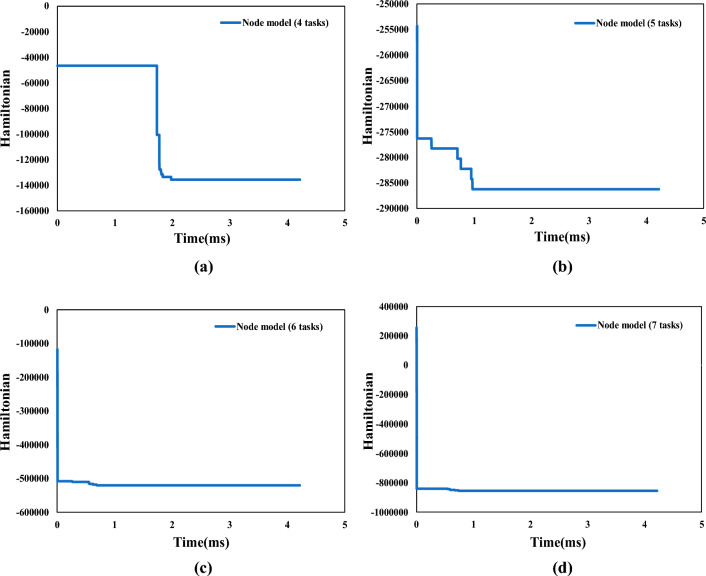
Evolution diagram of Hamiltonian with time under the objective functions of minimizing the total travel time in node model. (**a**) Represents the evolution diagram of Hamiltonian with time under the example of 4 tasks. (**b**) Represents the evolution diagram of Hamiltonian with time under the example of 5 tasks. (**c**) Represents the evolution diagram of Hamiltonian with time under the example of 6 tasks. (**d**) Represents the evolution diagram of Hamiltonian with time under the example of 7 tasks.

**Figure 5:**
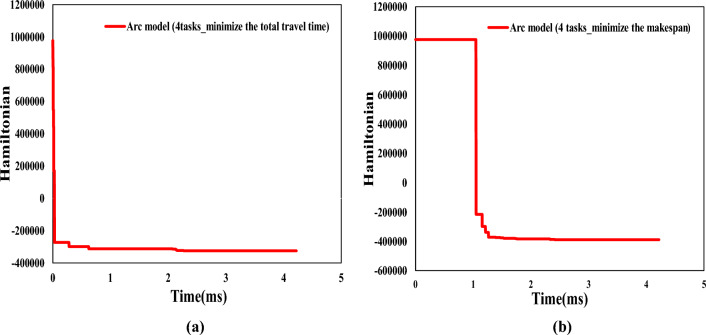
Evolution diagram of Hamiltonian with time under two objective functions of arc mode. (**a**) represents the evolution diagram of Hamiltonian with time under the objective function of minimizing the total travel time. (**b**) Represents the evolution diagram of Hamiltonian with time under the objective function of minimizing the makespan.

In Figs. 4 and  5, we plot the evolution of Ising Hamiltonian with time under node model and arc model, respectively. According to the above explanation of CIM principle construction, we can know that the time interval between every two adjacent data points in the figure is 2.11 microseconds. The Hamiltonian decreases with the passage of time, and the phase transition occurs as the power of the pump light gradually increases to the oscillation threshold. The solution obtained when reaching the lowest energy state is the result of CIM solution, and the corresponding time at this time is the computation time.

**Figure 6:**
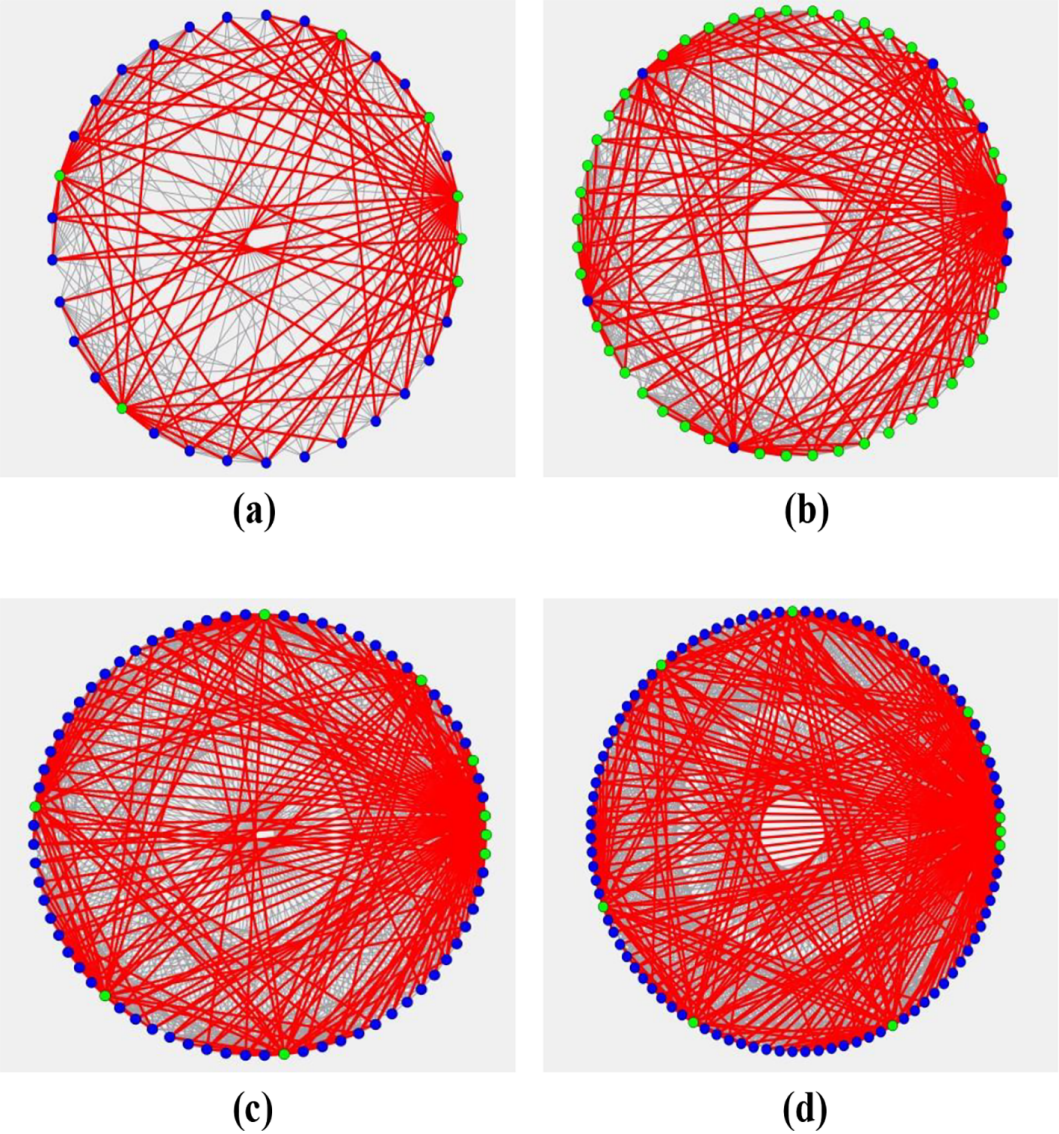
Schematic diagram of quantum computing solutions for node model. (**a**) represents solution under 4 tasks. (**b**) represents solution under 5 tasks. (**c**) represents solution under 6 tasks. (**d**) represents solution under 7 tasks.

**Figure 7:**
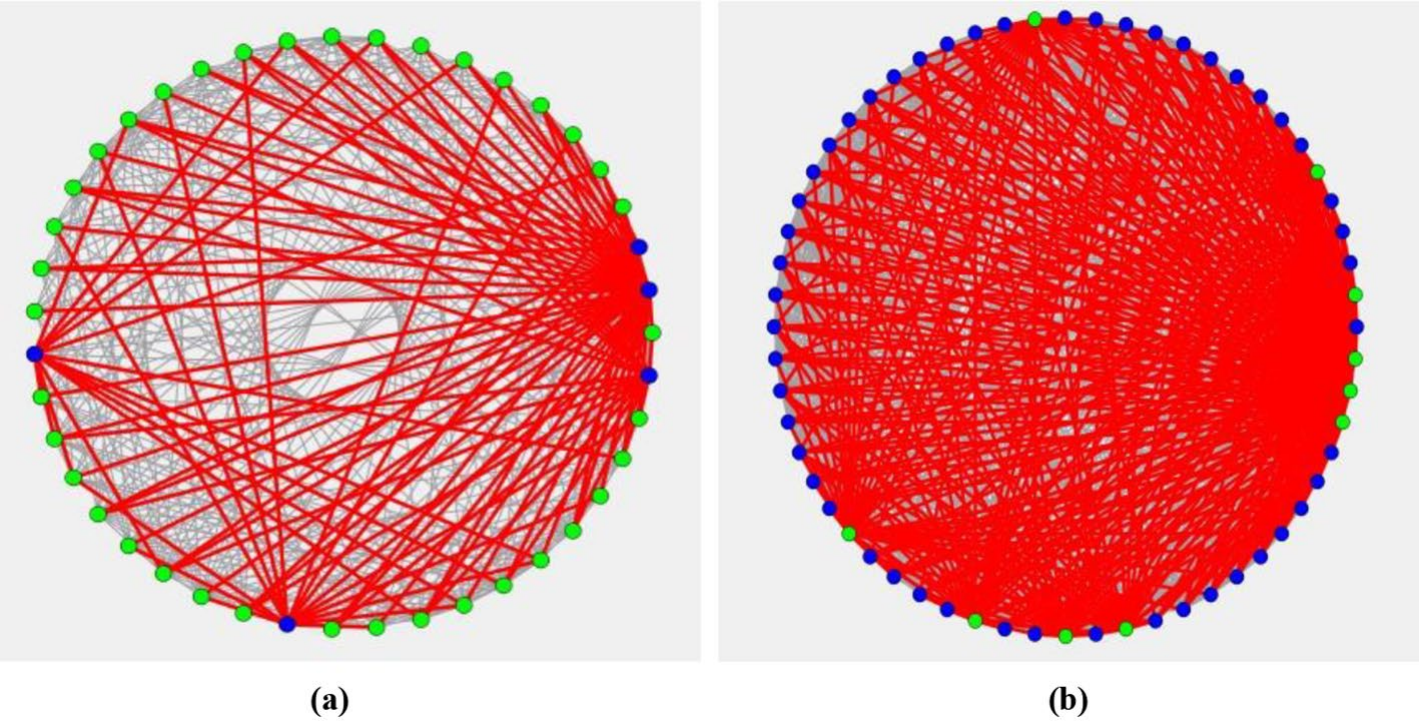
Schematic diagram of quantum computing solutions for arc model. (**a**) represents the solution of 4 task under the objective function of minimizing the total time. (**b**) represents the solution of 4 task under the objective function of minimizing the makespan.

Figures 6 and 7 show the schematic diagrams of CIM’s solution under node model and arc model. Among them, Fig. 6 contains four parts, which respectively represent the schematic diagrams under 4-7 tasks, while Fig. 7 contains two parts, which respectively represent the schematic diagrams of solving two objective functions under 4 tasks. In fact, Ising model can be transformed into the corresponding representation of maximum cut problem^15^. The maximum cut problem is usually used as a measure and demonstration basis for the complexity of quantum computing problems and the distribution of solutions. The figure shows the solution of our problem expressed by the representation method of maximum cut problem. Points with different colors indicate that they are in different groups, and the connecting lines of points in the same group are gray, while those of points in different groups are red. In these figures, we can clearly perceive the complexity of each model at different scales.

Here, we compare the performance of node model and arc model on quantum computer with that of mixed integer programming model on traditional computer, and the comparison results are shown in Table 3. An instance with *a* tasks and *b* AGVs are denoted by “a–b”.

**Table 3:** Computation time (ms) of traditional computer and CIM.

Instances	Objective	Mip (solved by traditional computer)		Node (solved by CIM)		Arc (solved by CIM)	
		Time	Obj	Time	Obj	Time	Obj
4–2	Total travel time	15.6	52.82	2.00	52.82	2.25	52.82
5–2	Total travel time	31.2	57.77	1.00	57.77	–	–
6–2	Total travel time	93.7	60.11	0.75	60.11	–	–
7–2	Total travel time	234.3	65.35	0.73	65.35	–	–
4–2	Makespan	15.6	44	–	–	2.30	44

Due to the limitation of hardware, the comparison of large-scale examples cannot be carried out. However, from Table 3, we can see that the solutions obtained by CIM are all optimal solutions. And the CIM is much faster than the traditional computer in small-scale examples. We can observe that CIM has obvious performance advantages over traditional computers in small-scale examples. In particular, when the scale increases, the time required for CIM does not increase significantly as that of traditional computers. This shows that CIM has great development and application potential. In addition, there is little difference in computing performance between node model and arc model on quantum computer. Node model is slightly faster than arc model, but arc model is more universal than node model. In order to measure the improvement of CIM’s computing efficiency compared with the traditional computer in the given example, we propose the following computation formula.

53 $$\begin{aligned} IMP=\frac{Q_{TRA}-Q_{CIM}}{Q_{TRA}} \end{aligned}$$

*IMP* represents the calculation speed improvement rate, $Q_{TRA}$ and $Q_{CIM}$ respectively represent the computation time of traditional computer and CIM on the same example, and both node model and arc model participate in the comparison. After calculating the *IMP* of all examples, we find that the computation efficiency of CIM is $92\%$ faster than that of traditional computers.

## Conclusion and future research

We applied quantum computing technology to the research on AGV scheduling, and proposed QUBO models that adapts to solve the problem under two different criteria, minimizing total AGV travel time and makespan. Compared with the traditional MIP model, numerical experiments were carried out on traditional computers and CIM. The experimental results proved the superiority and great potential of quantum computing in this field. Of course, due to the limitation of hardware, there are still some shortcomings in this study, which can not show the advantages of quantum computing in large-scale situations. It is believed that with the continuous development of quantum computing technology, the outstanding performance of quantum computing will be demonstrated in solving large-scale problems in the future. In addition, we also summarized the situation that this study can expand, as follows.

First, the model can be improved and expanded. The model we considered above applies to AGVs with a uniform start node and a uniform end node. Realistic scenarios exist where AGVs have different start and end nodes, and our model is easily expandable for these types of cases. The solution is to involve the number of virtual start and end tasks based on the number of AGVs. Due to the difficulty of mapping large-scale optimization problems to the QUBO form with the small number of bits currently available for quantum computing, our proposed model is a pure scheduling problem that does not consider path optimization. Of course, subsequent researchers can consider this possibility in the case of mature technology. We believe that a two-layer planning model can be built on the basis of the existing model, where the scheduling and path planning problems are computationally solved alternately in two sub-models, which reduces both the modeling difficulty and the number of bits used in the solution of a single model.

Second, quantum computer and traditional computer can be combined to study AGV scheduling problem, and their respective characteristics can be better used to improve the efficiency of solving this problem.

### Supplementary Information

Supplementary Information 1.

Supplementary Information 2.

Supplementary Information 3.

## Data Availability

The datasets used and/or analysed during the current study available from the corresponding author on reasonable request.

## Abbreviations

AGVs: Automated guided vehicles
QUBO: Quadratic uncontrained binary optimization
CIM: Coherent Ising machine
TSP: Traveling salesman problem
MIP: Mixed integer programming
FPGA: Field programmable gate array
EDFA: Erbium-doped fiber amplifier
PSA: Phase sensitive amplifier
DOPO: Degenerate optical parametric oscillation
BHD: Balanced homodyne detectors

## References

[1] Qin H (2022). Jd.com: Operations research algorithms drive intelligent warehouse robots to work. INFORMS J. Appl. Anal..

[2] Singh N, Dang Q-V, Akcay A, Adan I, Martagan T (2022). A matheuristic for AGV scheduling with battery constraints. Eur. J. Oper. Res..

[3] Zhang X-J, Sang H-Y, Li J-Q, Han Y-Y, Duan P (2022). An effective multi-AGVs dispatching method applied to matrix manufacturing workshop. Comput. Ind. Eng..

[4] Zhang L, Yan Y, Hu Y, Ren W (2021). A dynamic scheduling method for self-organized AGVs in production logistics systems. Procedia CIRP.

[5] Wang Z, Zeng Q (2022). A branch-and-bound approach for AGV dispatching and routing problems in automated container terminals. Comput. Ind. Eng..

[6] Sagar KV, Jerald J (2022). Real-time automated guided vehicles scheduling with Markov decision process and double q-learning algorithm. Mater. Today Proc..

[7] Saidi-Mehrabad M, Dehnavi-Arani S, Evazabadian F, Mahmoodian V (2015). An ant colony algorithm (aca) for solving the new integrated model of job shop scheduling and conflict-free routing of AGVs. Comput. Ind. Eng..

[8] Ajagekar A, Humble T, You F (2020). Quantum computing based hybrid solution strategies for large-scale discrete-continuous optimization problems. Comput. Chem. Eng..

[9] Yamamoto Y (2017). Coherent ising machines-optical neural networks operating at the quantum limit. NPJ Quant. Inf..

[10] Bunyk PI (2014). Architectural considerations in the design of a superconducting quantum annealing processor. IEEE Trans. Appl. Supercond..

[11] Wang Z, Marandi A, Wen K, Byer RL, Yamamoto Y (2013). Coherent ising machine based on degenerate optical parametric oscillators. Phys. Rev. A.

[12] Marandi A, Wang Z, Takata K, Byer RL, Yamamoto Y (2014). Network of time-multiplexed optical parametric oscillators as a coherent ising machine. Nat. Photon..

[13] McMahon PL (2016). A fully programmable 100-spin coherent ising machine with all-to-all connections. Science.

[14] Inagaki T (2016). A coherent ising machine for 2000-node optimization problems. Science.

[15] Honjo T (2021). 100,000-spin coherent ising machine. Sci. Adv..

[16] Lu B, Fan C-R, Liu L, Wen K, Wang C (2023). Speed-up coherent ising machine with a spiking neural network. Opt. Express.

[17] Lu B, Liu L, Song J-Y, Wen K, Wang C (2023). Recent progress on coherent computation based on quantum squeezing. AAPPS Bull..

[18] Aonishi T, Mimura K, Okada M, Yamamoto Y (2022). L0 regularization-based compressed sensing with quantum-classical hybrid approach. Quant. Sci. Technol..

[19] Takabatake K, Yanagisawa K, Akiyama Y (2022). Solving generalized polyomino puzzles using the ising model. Entropy.

[20] Osaba, E., Villar-Rodriguez, E. & Oregi, I. A systematic literature review of quantum computing for routing problems. *IEEE Access* (2022).

[21] Goswami, D., Karnick, H., Jain, P. & Maji, H. K. Towards efficiently solving quantum traveling salesman problem. http://arxiv.org/abs/quant-ph/0411013 (2004).

[22] Feld S (2019). A hybrid solution method for the capacitated vehicle routing problem using a quantum annealer. Front. ICT.

[23] Bao, S., Tawada, M., Tanaka, S. & Togawa, N. An approach to the vehicle routing problem with balanced pick-up using ising machines. in *2021 International Symposium on VLSI Design, Automation and Test (VLSI-DAT)*, 1–4 (IEEE, 2021).

[24] Harwood S (2021). Formulating and solving routing problems on quantum computers. IEEE Trans. Quant. Eng..

[25] Geitz, M., Grozea, C., Steigerwald, W., Stöhr, R. & Wolf, A. Solving the extended job shop scheduling problem with AGVs: Classical and quantum approaches. in *Integration of Constraint Programming, Artificial Intelligence, and Operations Research*, 120–137 (Springer, 2022).

[26] Ohzeki M, Miki A, Miyama MJ, Terabe M (2019). Control of automated guided vehicles without collision by quantum annealer and digital devices. Front. Comput. Sci..

[27] Dang Q-V, Singh N, Adan I, Martagan T, van de Sande D (2021). Scheduling heterogeneous multi-load AGVs with battery constraints. Comput. Oper. Res..

[28] Hu Y, Yang H, Huang Y (2022). Conflict-free scheduling of large-scale multi-load AGVs in material transportation network. Transp. Res. E.

[29] Murakami K (2020). Time-space network model and milp formulation of the conflict-free routing problem of a capacitated AGV system. Comput. Ind. Eng..

[30] Ising E (1925). Contribution to the theory of ferromagnetism. Z. Phys..

[31] Nabors C, Yang S, Day T, Byer R (1990). Coherence properties of a doubly resonant monolithic optical parametric oscillator. JOSA B..

[32] Marandi A, Leindecker NC, Pervak V, Byer RL, Vodopyanov KL (2012). Coherence properties of a broadband femtosecond mid-ir optical parametric oscillator operating at degeneracy. Opt. Express.

[33] Nannicini G (2019). Performance of hybrid quantum-classical variational heuristics for combinatorial optimization. Phys. Rev. E.

[34] Wen J (2023). Optical experimental solution for the multiway number partitioning problem and its application to computing power scheduling. Sci. Chin. Phys. Mech. Astron..

[35] Rodríguez-Heck E (2018). Linear and quadratic reformulations of nonlinear optimization problems in binary variables. 4OR.

[36] Chao Yang. *Data set. Figshare* (2024). Accessed 01 Mar 2024.

[37] Gurobi Optimization, LLC. Gurobi Optimizer Reference Manual (2023).

[38] Huang, Y. *et al.* Quantum computing for mimo beam selection problem: Model and optical experimental solution. http://arxiv.org/abs/2310.12389 (2023).

