# Supplementary material for: Quantum computing for several AGV scheduling models
Source: Sci Rep. 2024 May 28;14:12205. doi: 10.1038/s41598-024-62821-6 (PMC11133359; doi:10.1038/s41598-024-62821-6)
Supplement: Supplementary file 1 — Supplementary Information 1. [file 41598_2024_62821_MOESM1_ESM.docx]

Supplementary material information

1.Iterative data of quantum computer.xlsx

Abstract: This file contains the data of Hamiltonian variation with time and the Hamiltonian variation diagram drawn from this data.

2.Time matrix data between tasks.pdf

Abstract: This file is the task matrix used in the numerical experiment part of the article.
