# Supplementary material for: Quantum computing for several AGV scheduling models
Source: Sci Rep. 2024 May 28;14:12205. doi: 10.1038/s41598-024-62821-6 (PMC11133359; doi:10.1038/s41598-024-62821-6)
Supplement: Supplementary file 3 — Supplementary Information 3. [file 41598_2024_62821_MOESM3_ESM.pdf]

This dataset contains two parts of time, the first part is the time from the start of task r to the end of task r, and the second part is the time from the end of task r to the start of task r′, The range is 4 tasks to 13 tasks. The figures in the 4 task examples are all integers for the convenience of quantum computer calculation.

```
#13tasks
c=np.array([[1000,18.68,16.12,15.13,16,20.1,38.07,39.36,40.3
1,33.36,10,12.17,19.65],

[1000,1000,7,7.83,8.4,9,27,29.46,31.15,32.41,27,26.76,12.05]
,

[1000,5,1000,5,4.83,6.47,26.04,28.25,29.8,29.89,23.63,23.26,
9.07],

[1000,9.56,8.08,1000,7.47,6.71,26.41,28.56,30.09,30.19,25.95
,25.29,9.47],

[1000,9.83,7.3,7.83,1000,4.83,24.23,26.18,27.59,26.87,22.53,
21.7,5.99],

[1000,15.2,12,12.07,10,1000,26.47,24.1,29.21,26.93,21.76,20.
65,8],
[1000,34.48,32.37,32.8,30.56,27.2,1000,20.44,20.63,17.44,36.
69,34.21,24.76],

[1000,33.12,31.46,32.02,29.8,25.98,12.46,1005.39,10.39,12.46
,37.01,34.51,23.99],

[1000,38.33,36.07,36.44,34.21,31.06,22.65,21.21,1000,13.44,3
8.17,35.52,28.47],

[1000,37.31,35.41,35.89,33.65,30.08,16.18,13.6,12.07,1000,38
.54,35.87,27.83],

[1000,23.93,20.44,19.69,19.2,21.7,35.38,35.96,36.48,29.45,10
00,4,19.72],

[1000,24.4,20.8,20.2,19.28,21.03,33.15,33.59,34.05,26.85,8.3
8,1000,18.65],

[1000,1000,1000,1000,1000,1000,1000,1000,1000,1000,1000,1000
```

```
,1000]])
#12tasks
c=np.array([[1000,18.68,16.12,15.13,16,20.1,38.07,39.36,40.3
1,33.36,10,19.65],
 [1000,1000,7,7.83,8.4,9,27,29.46,31.15,32.41,27,12.05],

[1000,5,1000,5,4.83,6.47,26.04,28.25,29.8,29.89,23.63,9.07],

[1000,9.56,8.08,1000,7.47,6.71,26.41,28.56,30.09,30.19,25.95
,9.47],

[1000,9.83,7.3,7.83,1000,4.83,24.23,26.18,27.59,26.87,22.53,
5.99],

[1000,15.2,12,12.07,10,1000,26.47,24.1,29.21,26.93,21.76,8],
[1000,34.48,32.37,32.8,30.56,27.2,1000,20.44,20.63,17.44,36.
69,24.76],

[1000,33.12,31.46,32.02,29.8,25.98,12.46,1005.39,10.39,12.46
,37.01,23.99],

[1000,38.33,36.07,36.44,34.21,31.06,22.65,21.21,1000,13.44,3
8.17,28.47],

[1000,37.31,35.41,35.89,33.65,30.08,16.18,13.6,12.07,1000,38
.54,27.83],

[1000,23.93,20.44,19.69,19.2,21.7,35.38,35.96,36.48,29.45,10
00,19.72],

[1000,1000,1000,1000,1000,1000,1000,1000,1000,1000,1000,1000
]])
#11tasks
c=np.array([[1000,18.68,16.12,15.13,16,20.1,38.07,39.36,40.3
1,33.36,19.65],
 [1000,1000,7,7.83,8.4,9,27,29.46,31.15,32.41,12.05],
 [1000,5,1000,5,4.83,6.47,26.04,28.25,29.8,29.89,9.07],

[1000,9.56,8.08,1000,7.47,6.71,26.41,28.56,30.09,30.19,9.47]
,

[1000,9.83,7.3,7.83,1000,4.83,24.23,26.18,27.59,26.87,5.99],
 [1000,15.2,12,12.07,10,1000,26.47,24.1,29.21,26.93,8],
[1000,34.48,32.37,32.8,30.56,27.2,1000,20.44,20.63,17.44,24.
```

```
76],

[1000,33.12,31.46,32.02,29.8,25.98,12.46,1005.39,10.39,12.46
,23.99],

[1000,38.33,36.07,36.44,34.21,31.06,22.65,21.21,1000,13.44,2
8.47],

[1000,37.31,35.41,35.89,33.65,30.08,16.18,13.6,12.07,1000,27
.83],
 [1000,1000,1000,1000,1000,1000,1000,1000,1000,1000,1000]])
#10tasks
c=np.array([[1000,18.68,16.12,15.13,16,20.1,38.07,39.36,40.3
1,19.65],
 [1000,1000,7,7.83,8.4,9,27,29.46,31.15,12.05],
 [1000,5,1000,5,4.83,6.47,26.04,28.25,29.8,9.07],
 [1000,9.56,8.08,1000,7.47,6.71,26.41,28.56,30.09,9.47],
 [1000,9.83,7.3,7.83,1000,4.83,24.23,26.18,27.59,5.99],
 [1000,15.2,12,12.07,10,1000,26.47,24.1,29.21,8],
[1000,34.48,32.37,32.8,30.56,27.2,1000,20.44,20.63,24.76],

[1000,33.12,31.46,32.02,29.8,25.98,12.46,1005.39,10.39,23.99
],

[1000,38.33,36.07,36.44,34.21,31.06,22.65,21.21,1000,28.47],
 [1000,1000,1000,1000,1000,1000,1000,1000,1000,1000]])
#9tasks
c=np.array([[1000,18.68,16.12,15.13,16,20.1,38.07,39.36,19.6
5],
 [1000,1000,7,7.83,8.4,9,27,29.46,12.05],
 [1000,5,1000,5,4.83,6.47,26.04,28.25,9.07],
 [1000,9.56,8.08,1000,7.47,6.71,26.41,28.56,9.47],
 [1000,9.83,7.3,7.83,1000,4.83,24.23,26.18,5.99],
 [1000,15.2,12,12.07,10,1000,26.47,24.1,8],
[1000,34.48,32.37,32.8,30.56,27.2,1000,20.44,24.76],

[1000,33.12,31.46,32.02,29.8,25.98,12.46,1005.39,23.99],
 [1000,1000,1000,1000,1000,1000,1000,1000,1000]])
#8tasks
c=np.array([[1000,18.68,16.12,15.13,16,20.1,38.07,19.65],
 [1000,1000,7,7.83,8.4,9,27,12.05],
 [1000,5,1000,5,4.83,6.47,26.04,9.07],
 [1000,9.56,8.08,1000,7.47,6.71,26.41,9.47],
 [1000,9.83,7.3,7.83,1000,4.83,24.23,5.99],
```

```
 [1000,15.2,12,12.07,10,1000,26.47,8],
[1000,34.48,32.37,32.8,30.56,27.2,1000,24.76],
 [1000,1000,1000,1000,1000,1000,1000,1000]])
#7tasks
c=np.array([[1000,18.68,16.12,15.13,16,20.1,19.65],
 [1000,1000,7,7.83,8.4,9,12.05],
 [1000,5,1000,5,4.83,6.47,9.07],
 [1000,9.56,8.08,1000,7.47,6.71,9.47],
 [1000,9.83,7.3,7.83,1000,4.83,5.99],
 [1000,15.2,12,12.07,10,1000,8],
 [1000,1000,1000,1000,1000,1000,1000]])
#6tasks
c=np.array([[1000,18.68,16.12,15.13,16,19.65],
 [1000,1000,7,7.83,8.4,12.05],
 [1000,5,1000,5,4.83,9.07],
 [1000,9.56,8.08,1000,7.47,9.47],
 [1000,9.83,7.3,7.83,1000,5.99],
 [1000,1000,1000,1000,1000,1000]])
#5tasks
c=np.array([[1000,18.68,16.12,15.13,19.65],
 [1000,1000,7,7.83,12.05],
 [1000,5,1000,5,9.07],
 [1000,9.56,8.08,1000,9.47],
 [1000,1000,1000,1000,1000]])
#4tasks
c=np.array([[100,12,16,3],
 [100,100,27,32],
 [100,24,100,28],
 [100,100,100,100]])
```
